# Delay in diagnosis and treatment of childhood cancers in a developing country: A cross-sectional patient-based survey at country’s largest pediatric oncology center

**DOI:** 10.12669/pjms.41.5.9663

**Published:** 2025-05

**Authors:** Rahat Ul Ain, Ali Abdur Rahman, Asfand Tariq, Mahwish Faizan

**Affiliations:** 1 Rahat Ul Ain, MBBS, FCPS (Pediatric Medicine), FCPS (Pediatric Hematology/Oncology) Assistant Professor, Department of Pediatric, Hematology-Oncology and Bone Marrow Transplant, University of Child Health Sciences, The Children’s Hospital, Ferozepur Road, Lahore, Pakistan; 2 Ali Abdur Rahman, MBBS Postgraduate resident in Pediatric Medicine, Shaikh Zayed Hospital Lahore, Lahore, Pakistan; 3 Asfand Tariq, MBBS, FCPS (Pediatric Medicine) Assistant Professor, Department of Pediatric Medicine, Shaikh Zayed Hospital Lahore, Lahore, Pakistan; 4 Mahwish Faizan Associate Professor, Department of Pediatric Hematology, Oncology and Bone Marrow Transplant, University of Child Health Sciences, The Children’s Hospital, Ferozepur Road, Lahore, Pakistan

**Keywords:** Developing country, Delayed diagnosis, Factors, Referral, Lag-time, Oncology, Pediatric

## Abstract

**Background & Objectives:**

Pediatric cancers have a huge survival gap between the high-income and the low-middle-income countries (LMICs). Despite various other causes, the advanced stage of presentation is a major contributing factor but limited data is available from developing countries in this regard. This study aimed to identify delay in treatment and factors associated with it in our setup.

**Methods:**

This was a Cross-Sectional survey conducted at the department of pediatric hematology-oncology Children Hospital Lahore using a questionnaire-based interview of parents of all newly diagnosed cases of pediatric cancers presenting to Pakistan’s largest public-sector specialized center at the Children’s Hospital Lahore, over two months, i.e. from December 1, 2023 to January 31, 2024. The data was analyzed using SPSS version 23.0.

**Results:**

A total of 119 new patients presenting to the hospital were included. The median total delay in treatment of children with cancer was 63 days which is less than comparable LMICs, but physician and healthcare system delay that makes up the major portion of the total delay is equivalent to some low-income countries. The patient delay was negligible. The statistically significant factors associated with delay were gender, age of the patient; and type of cancer. Whereas, the distance from the specialized center and the socio-economic status of the family did not contribute significantly.

**Conclusions:**

Healthcare system delay is a major contributor therefore strengthening the existing medical services, development of medical referral systems, and medical professional awareness and education can improve delays in treatment and outcomes in our setup.

## INTROUCTION

Each year, approximately 400,000 children and adolescents aged 0-19 years are expected to develop cancer, out of which > 90% are expected from low and middle-income countries, where the majority receive suboptimal medical care due to poor healthcare systems.^1^ The estimated five years survival rate surpasses 80% for high-income countries (HICs), which are estimated to be less than 30% in low (LICs) and lower-middle-income countries (LMICs).^2^ Even though cancer is a rare diagnosis in children, it remains a major cause of childhood mortality, with an estimated 80,000 deaths per year worldwide, most of which occur in LMICs.^3^,^4^

**Fig.1:**
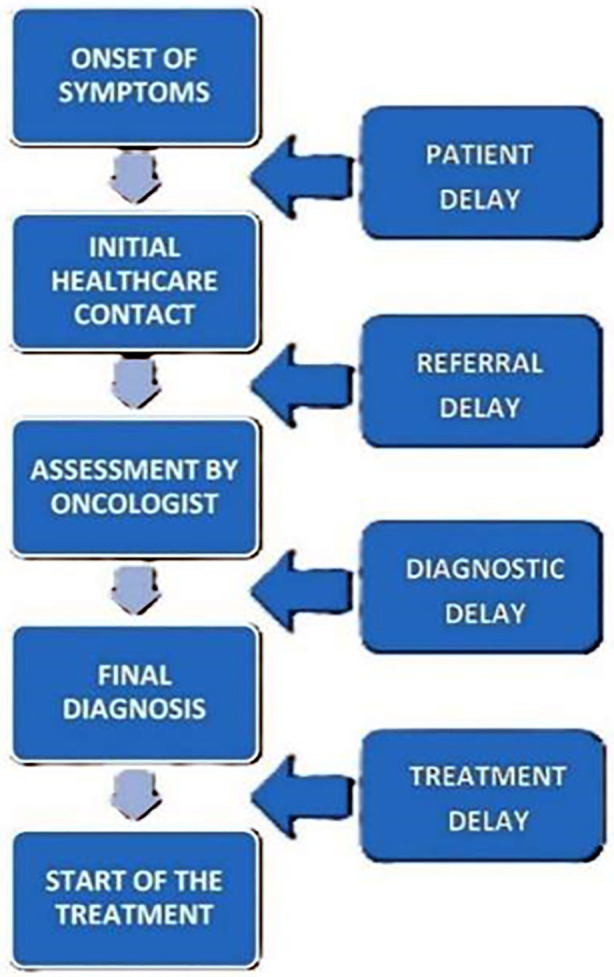
Schematic diagram representing the delay in management of cancer from onset of symptoms to treatment initiation.

About 7000 to 7500 children get cancer yearly in Pakistan.^5^ Despite other well-known disease prognostic factors, delay in the treatment is a major contributing prognostic factor in LICs/LMICs. The total delay in treatment encompasses delay at three major steps of care and management:

- Delay in presentation to primary care physician (due to patient negligence/lack of awareness)
- Delay in referral to oncological center (due to inadequate primary care physician skills, and inadequate knowledge about the available specialized facilities in the region)
- Delay in final diagnosis and definitive treatment (due to patient load/limited diagnostic facilities).^6^,^7^ (Fig.1)
- An effective assessment of causal factors followed by targeted interventions is needed to improve treatment delays. This study aimed to identify the lag time in presentation, reasons, and factors associated with delays in our setup, and compare the statistics with the studies conducted in other countries.

## METHODS

It was a Cross-Sectional study conducted using a Questionnaire-based interview with the parents of the eligible patients presenting to the Department of Pediatric Hematology/Oncology, at The Children’s Hospital Lahore, over two month, i.e. from 1^st^ December 2023 to 31^st^ January 2024. This center has an inpatient 60 bedded facility and is Pakistan’s largest, public-sector, pediatric oncology center that offers free-of-cost treatment to > 1500 new patients/year from all over the country, and neighboring country Afghanistan. All of our patients were under the age of 18 years, male to female ratio was 2:1 (80 males to 39 females), and 96.6% (115/119) of participants had a family income under PKR 50,000 with only 3.4% (4/119) having a family income PKR 50,000 to 175,000. The questionnaire was created by Dr. Rahat-Ul-Ain and included age of patient, gender, family income, literacy status of head of family, number of siblings, area of residence, diagnosis of disease, date of presentation to primary care physician, date of presentation to oncological center, date of presentation to our oncology center, total duration since appearance of first symptoms and signs of disease, and cause of delay according to the parents.

### Data collection:

All children aged ≤ 18 years, newly admitted with a suspected or confirmed diagnosis of cancer were included in the study. Patients already on treatment or on follow-up were excluded.

### Ethical Approval:

The study was approved by the institutional ethical committee (No./734/CH-UCHS dated November 15, 2023).

After informed consent, the parents/guardians were interviewed, and all relevant clinical documents (referral reports, laboratory tests, and prescriptions) were reviewed to document the date of first medical contact, subsequent referrals, visits to any oncological center and the date of presentation to our center. An estimated time in weeks was calculated and noted. Information regarding age, gender, the status of disease (suspected or diagnosed case of cancer), diagnosis, area of residence, and socio-economic status of the family was also recorded maintaining anonymity and confidentiality.

### Data analysis:

The data was analyzed in the SPSS version 23.0. The numerical data was presented as median and interquartile range (IQR) (25^th^-75^th^ centiles) as not normally distributed. The total delay was the dependent variable and was compared with the independent variables with two subsets using the Mann-Whitney test while the comparison with >2 subsets independent variables was done using the Kruskal-Wallis test. The P value ≤ 0.050 was considered as statistically significant.

### Operational definitions:

### Total delay:

Defined as the total time in weeks from the onset of patient’s symptoms to the presentation at the oncological center, time to diagnosis and beginning of definitive treatment.

### Patient/Parent delay:

Time in weeks from disease onset to visiting the primary care physician.

### Referral delay:

Time in weeks from visiting the first physician to the time in getting referred to an oncology center.

### Diagnostic delay:

Time in weeks from presenting to specialized center to definitive diagnosis.

### Treatment delay:

Time in weeks from diagnosis to start of definitive treatment.

### Physician delay:

Defined as a sum of diagnostic and referral delay.

### Healthcare system delay:

Defined as a total of referral delay, diagnostic delay and treatment delay.

## RESULTS

A total of 119 new patients presented over one month, with an age range of four months to 17 years, and a male-to-female ratio of 2:1. Most of the patients were from outside Lahore including Punjab, Khyber Pakhtunkhwa, Sindh (including two from Afghanistan). They belonged to low socioeconomic background with 96.6% having a family income under PKR 50,000 and only 3.4% having an income greater than PKR 50,000 – 175,000. According to literacy rate of head of family, 42.9% of participants were illiterate, 16.0% had primary education, 12.6% had middle education, 19.3% had matric, 5.9% had intermediate, 2.5 % were graduates and only 1 participant with masters education. The response rate to questioned patients was 100% and there were no reported refusals.

**Fig.2:**
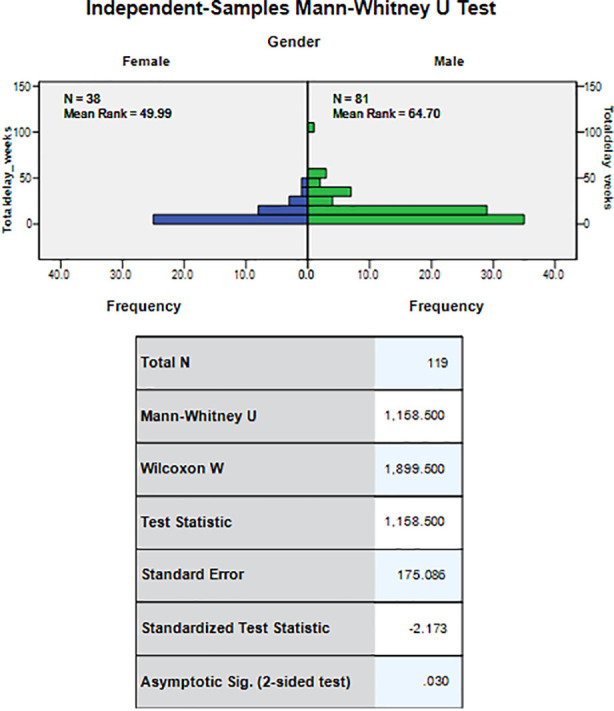
Relationship of gender with total delay.

**Fig.3:**
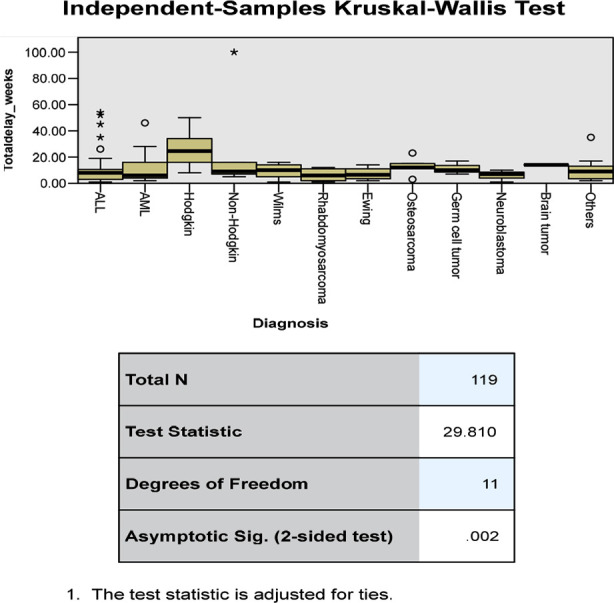
Relationship of type of cancer with total delay.

**Fig.4:**
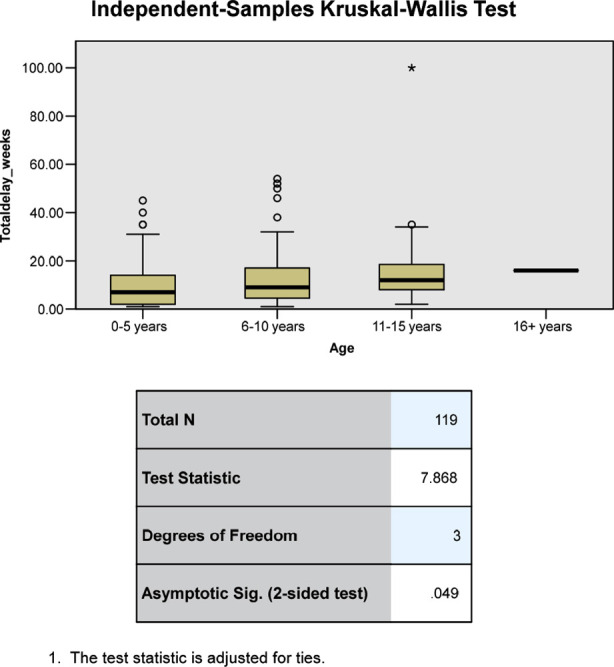
Relationship of age with total delay.

The Median total delay for all the patients was 9.0 weeks (Range 1-100 weeks), and the median patient delay was 0.0 weeks (Range 0-2 weeks), median referral delay was 7.0 weeks (Range 0-97 weeks), median diagnostic delay was 1.0 week (Range 0-8 weeks), median treatment delay 0.0 weeks (Range 0-20 weeks), median physician delay and the median healthcare system delay both were eight weeks each. The relationship of various factors to all types of delays is shown in Table-I & Table-II. The statistically significant factors found to be associated with total delay were gender, age of the patient; and type of cancer. (Fig.2,3, and 4).

**Table-I:** Median total delay in relation to patient characteristics.

Characteristic (INDEPENDENT)	No. Of patients (percentage %)	Median Total delay (WEEKS); (IQR 25th-75th) - (DEPENDENT)	P-value
***Gender:***			0.03
Female	38 (31.9%)	8.0 (4.0-12.0)	
Male	81 (68.1%)	11.0 (5.0-17.0)	
***Age (Years):***			0.049
0 - 5	44 (37.0%)	7.0 (2.0-14.0)	
6 - 10	47 (39.5%)	9.0 (4.5-17.0)	
11 - 15	27 (22.7%)	12.0 (8.0-20.0)	
16+	1 (0.8%)	-	
***Parents Education:***			0.786
Illiterate	51 (42.9%)	10.0 (4.0-16.5)	
Primary	19 (16.0%)	8.0 (4.5-16.0)	
Middle	15 (12.6%)	9.0 (3.5-24.5)	
Matric	23 (19.3%)	8.0 (7.0-14.5)	
Intermediate	07 (5.9%)	6.0 (3.0-12.0)	
Graduate	03 (2.5%)	16.0 (11.5-34.0)	
Masters	01 (0.8%)		
***Disease status:***		8.0 (4.0-18.5)	0.923
Diagnosed	67 (56.3%)	10.0 (5.0-16.0)	
Suspected	52 (43.7%)		
***Economic status:***			0.456
Low (<50,000 PKR/month)	115 (96.6%)	9.0 (5.0-16.0)	
Middle (50,000-175,000 PKR/month)	4 (3.4%	7.0 (3.0-13.0)	
High (> 175,000 PKR/month)	-	-	
***Type of Malignancy:***			0.002
ALL	36 (30.3%)	8.0 (3.0-10.5)	
AML	17 (14.3%)	6.0 (4.0-16.0)	
Hodgkin Lymphoma	18 (15.1%)	24.5 (16.0-34.25)	
Others	10 (8.4%)	9.0 (3.5-13.0)	
Non-Hodgkin Lymphoma	10 (8.4%)	9.0 (7.0-16.0)	
Wilms tumor	6 (5.0%)	10.0 (5.0-14.0)	
Rhabdomyosarcoma	5 (4.2%)	6.0 (2.0-11.0)	
Osteosarcoma	5 (4.2%)	12.0 (12.0-15.0)	
Germ cell tumor	4 (3.4%)	10.0 (8.5-13.5)	
Ewing sarcoma	4 (3.4%)	6.5 (3.5-11.0)	
Neuroblastoma	3 (2.5%)	7.0 (4.0-8.5)	
Brain tumor	1 (0.8%)	-	
***Location:***			0.726
Within 100km of Lahore	37 (31.1%)	10.0 (7.0-16.0)	
Outside 100km of Lahore	82 (68.9%)	8.5 (4.0-16.0)	

**Table-II:** Different delays with patient characteristics.

Characteristics	MEDIAN Patient delay (Weeks)	MEDIAN Referral delay (Weeks)	MEDIAN Diagnostic delay (Weeks)	MEDIAN Treatment delay (Weeks)
***Gender***				
Male	0	9	1	0
Female	0	5.5	1	0
***Age (YEARS)***				
0-5	0	4.5	1	0
6-10	0	7	1	0
11-15	0	9	1	0
16+	1	13	2	0
***Type of cancer***				
ALL	0	6	1	0
AML	0	4	1	0
Hodgkin Lymphoma	0	17.5	2	0
Others	0	5.0	1.5	0
Non-Hodgkin Lymphoma	0	7.0	2.0	0
Wilms tumor	0	8.0	1.0	0
Rhabdomyosarcoma	0	4.0	1.0	0
Osteosarcoma	2.0	9.0	2.0	0
Germ cell tumor	1.0	7.0	1.0	0
Ewing sarcoma	0	5.0	1.0	0
Neuroblastoma	0	1.0	1.0	0
Brain tumor	2.0	10.0	4.0	0
***Education***				
Illiterate	0	7	1	0
Up to Matric	0	7	1	0
High education	0	5	1	0
***Financial status***				
Low (<50,000 PKR/month)	0	7	1	0
Middle (50,000-175,000 PKR/month)	0	5.5	1	0
***Disease status:***				
Diagnosed	0	7	1	0
Suspected	0	7	1	0
***Distance***				
Within 100 km of Lahore	1	7	1	0
Outside 100 km of Lahore	0	7	1	0

## DISCUSSION

In this study, the most striking finding is the median physician delay which makes up a major portion of the total delay. Patient delay and treatment delays were not important contributors to the total delay. Factors at the individual, community, and systems levels may influence the access and timeliness of medical care.^8^ A significant difference in the lag time between the onset of symptoms and diagnosis exists based on country income level.^9^ In South Asia especially, childhood cancer is considerably underdiagnosed, and apart from improving treatments, strengthening of health systems is a major factor in the effectiveness of care of children with cancer.^10^ One similar study was conducted on pediatric cancer patients from Pakistan (Bano S et al.)^11^ showed other factors affecting the delay like misdiagnosis, lack of transportation and lack of awareness.

Another study from Pakistan (Rahat-Ul-Ain Kashif et al.)^12^ also highlighted that the most common reason for delayed presentation of children with malignant mediastinal masses was delayed diagnosis by medical professionals. Therefore, it can be inferred that Physician delay plays a substantial role in delays in cancer care in children in Pakistan. It is also observed that the physician delay observed in this study is much higher than similar studies conducted in comparable LMICs (Egypt, Indonesia, India, and Turkey)^3^,^7^,^13^-^15^ This might be due to many reasons, but a few major ones could be the physician shortage in rural area hospitals, patients overburden in urban areas hospitals, physician neglect of cancer symptoms due to rarity of disease, more healthcare concerns regarding nutritional deficiencies, and infectious diseases, rather than the non-communicable diseases like childhood cancers, and lack of diagnostic facilities in primary and secondary healthcare facilities.

In this study the male gender and adolescent age group were associated with noteworthy total delay and diagnostic delay however, the patient delay, and treatment delays were not significantly affected. The median total delay and median referral delay in females were lesser than that of males. This pattern is different than previous studies conducted in Egypt, Kenya, and Turkey where there was no effect of gender type on the delay times.^3^,^6^,^14^ It could be due to the small sample size of girls in our study, or probably because girls are keener on self-care and health as compared to boys.

The longest median total delay and median referral delay were seen in children in the adolescent age group. This correlates to the effects seen in previous studies in Egypt, Kenya, Indonesia, and Turkey.^3^,^6^,^7^,^14^ This could be due to the more aggressive nature of cancers in younger age groups leading to an earlier presentation, and as opposed to younger children where the parental observation is close leading to an earlier recognition of symptoms, children of older age groups might underestimate the signs and symptoms that they experience leading to increased delay.

### The median total delay:

In a similar study from Egypt 3 was much shorter than observed in our study while studies from Kenya^6^ and Indonesia^7^ show greater median total delay. Comparable median total delay is seen by Verma N et al. and Araz NC et al.^13^,^14^ *The median patient delay* observed in various studies from LIMCs was comparable to our study^3^,^6^-^8^ but much shorter than seen in a study from Turkey^14^, and from India.^15^ Such differing results could be due to differences in healthcare systems; social, economic, and cultural factors. A recent systematic review described traditional medicine, household income, lack of transportation, rural population, parental education, and travel distance as the determinants of delays in childhood cancer care in LMICs.^16^

Data regarding treatment delays in HICs reported that rural families faced increased travel time to their cancer centers and increased time spent in community hospitals. Moreover, a misconception that cancer is rare in children leads to physicians being less proactive in investigating cancer until it is overtly progressive, and cancers with a relatively indolent course and subtle symptoms are associated with the longest total diagnostic interval.^17^-^19^ In this study different types of cancers had a statistically significant effect on the total delay, referral delay, and diagnostic delay, however, they did not significantly affect the patient delay and the treatment delay. The shortest delay was seen in leukemia while the longest delay was seen in Hodgkin lymphoma and brain tumors. This reinforces the fact that cancers with indolent course or concealed symptoms are associated with delay.^3^,^6^,^7^,^13^,^14^,^18^

Interestingly in this study, the literacy and financial status of the family, and the distance from the oncology center did not have a statistically significant effect on the total delay, patient delay, referral delay, diagnostic delay or treatment delay, and unpredictably, the delay was longer in those residing within 100 km of Lahore. Contrary to this, similar studies from Ethiopia^20^ and India^21^ found such socio-economic factors as major contributory factors to delay.

### Limitations:

The short duration of the study, recall bias regarding the symptom initiation and point of first contact with the primary care physician, inadequate information in a few cases due to the lack of database available for medical records in our set up, language barrier (especially with patients from Afghanistan and Khyber Pakhtunkhwa), and social/cultural discomfort in providing few pieces of information.

### Strengths:

Compared to previous studies (mostly conducted at a semi-public/ regional center) our study was conducted in the largest public-sector pediatric oncology referral center in the country. Therefore, it is expected that this data may closely represent the population of childhood cancer in the country. This has highlighted the areas in our healthcare system that warrant considerable interventions to improve the outcome of children with cancer in Pakistan.

## CONCLUSION

In Pakistan’s largest public-sector pediatric oncology center, the median total delay in treatment of children with cancer is less than comparable LMICs, but physician delay that makes up the major portion of the total delay is similar to some LICs. Unexpectedly, patient delay did not contribute notably to the total delay.

The statistically significant factors found to be associated with the total delay were male gender, adolescent age, and cancers with insidious or concealed symptoms. Concrete steps must be taken by the administrative and governing authorities to improve medical referral systems, strengthen the human resources with skilled pediatric oncologists at tertiary care centers and general physicians at primary/secondary healthcare facilities, and run general public and medical professional awareness programs regarding signs and symptoms of various pediatric cancers and information of specialized centers offering free diagnostic and treatment facilities in the province and country.

### Author`s Contribution:

**RUL:** Conception, design, and interpretation of data, drafting and revision of the article.

**AAR:** Acquisition of data, analysis and interpretation, drafting and revision of the article.

**AT:** Conception and design, revision of the article.

**MF:** Conception design, and interpretation of data, critical revision.

All authors have approved final approval version to be published and are accountable for all aspects of the work.
